# Identification of Antioxidative Hydrolyzable Tannins in Water Chestnut

**DOI:** 10.3390/molecules28186563

**Published:** 2023-09-11

**Authors:** Takashi Uchikura, Yuka Miura, Morio Yoshimura, Hideyuki Ito, Yoshiaki Amakura

**Affiliations:** 1 Department of Pharmacognosy, College of Pharmaceutical Sciences, Matsuyama University, 4-2 Bunkyo-cho, Matsuyama 790-8578, Ehime, Japan; t.uchikura@g.matsuyama-u.ac.jp (T.U.); miyurayuka@gmail.com (Y.M.); myoshimu@g.matsuyama-u.ac.jp (M.Y.); 2 Department of Nutritional Science, Faculty of Health and Welfare Science, Okayama Prefectural University, 111 Kuboki, Soja 719-1197, Okayama, Japan; hito@fhw.oka-pu.ac.jp

**Keywords:** water chestnut, *Trapa* genus, hydrolyzable tannin, trapadin A, antioxidant activity

## Abstract

Despite the various biological activities exhibited by water chestnut (the fruit of the *Trapa* genus), the phenolic compounds present in its extract require comprehensive characterization. Accordingly, we analyzed a 80% methanol extract of commercially available water chestnut and identified a new hydrolyzable tannin dimer termed trapadin A. Additionally, 22 known compounds, including 10 hydrolyzable tannin monomers and 2 dimers, were also detected in the extract. Spectroscopic and chemical methods were used to elucidate the structure of trapadin A, revealing it to be a hydrolyzable tannin dimer formed from units of tellimagrandin II and 1,2,3,6-tetra-*O*-galloyl-β-d-glucose. Moreover, the 1,1-diphenyl-2-picrylhydrazyl radical scavenging activity assay used to determine the half-maximal effective concentration values for the 23 compounds isolated from water chestnut indicated significant radical scavenging activity associated with hydrolyzable tannins. Notably, trapadin A, the new hydrolyzable tannin dimer, exhibited the highest activity value among the tested compounds.

## 1. Introduction

The water chestnut is the fruit of the aquatic plant *Trapa* genus, which includes several species such as *T. japonica*, *T. incisa*, and *T. bispinosa*. It thrives in swamps and lakes across Asia and features a hard walnut-like shell. In Japan, particularly in the Kyushu region, the edible starchy portion of the water chestnut is boiled and consumed along with the husks, yielding a tea that is considered to have stomachic and nourishing effects [1]. Notably, water chestnut primarily consists of carbohydrates, with lipids, proteins, and carbohydrates constituting 0.5, 5.8, and 40.6 g per 100 g, respectively. Additionally, this fruit is rich in vitamin B1 and folic acid [2]. Water chestnuts from *T. japonica* and *T. bispinosa* also reportedly contain hydrolyzable tannins [1,3,4,5]. Biological studies have indicated diverse health benefits associated with water chestnut, including antioxidant [1,6], antidiabetic [5,7,8], anti-obesity [9,10], anti-glycation [5], anti-inflammatory [11,12], antiadipogenic [6], and hepatic protective effects [13]. Furthermore, it has demonstrated inhibitory activity against α-amylase [14] and α-glucosidase [3,5,14]. Recently, water chestnut fruit extract has garnered attention for its potential in skin improvement [15] and as a treatment for alopecia [16], positioning it as a promising cosmeceutical and beauty food.

Considering the presence of diverse polyphenolic compounds in water chestnuts that exert various biological activities, a comprehensive investigation to identify its characteristic phenolic compounds is warranted. Moreover, antioxidant function is a typical biological activity of polyphenols [17]. Antioxidants in foods contribute to the maintenance of health and reduce the risk of various age-related diseases [18]. The contribution of their activity has been attributed to their chemical structure [17]. Therefore, detailed structural elucidation of the polyphenol activity in foods is important.

Accordingly, we isolated, identified, and characterized phenolic compounds from commercially available water chestnuts. Additionally, 1,1-diphenyl-2-picrylhydrazyl (DPPH) radical scavenging activity assays were performed for the obtained compounds.

## 2. Results and Discussion

### 2.1. Isolation and Characterization of Phenolic Constituents

**Figure 1:**
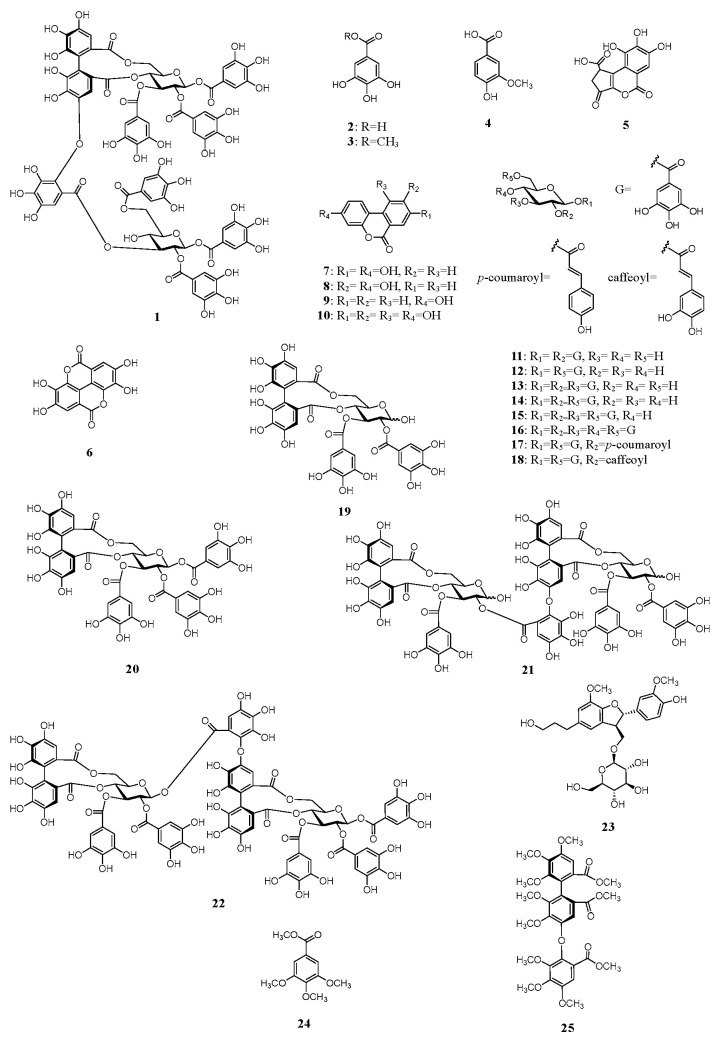
Structures of compounds **1**–**25**.

An 80% aqueous methanol (MeOH) extract of whole water chestnut was concentrated to obtain a water (H_2_O) extract. The H_2_O extracts were separately chromatographed using Diaion HP-20, YMC GEL ODS-AQ, MCI-gel CHP-20P, and Sephadex LH-20 with MeOH–H_2_O or ethanol (EtOH)–MeOH in a stepwise gradient mode. The fractions with similar high-performance liquid chromatography (HPLC) chromatograms were merged and further purified using column chromatography to obtain trapadin A (**1**), gallic acid (**2**) [19], methyl gallate (**3**) [19], vanillic acid (**4**) [19], brevifolincarboxylic acid (**5**) [20], ellagic acid (**6**) [21], urolithin A (**7**) [22], isourolithin A (**8**) [13], urolithin B (**9**) [11], urolithin M6 (**10**) [22], 1,2-di-*O*-galloyl-β-d-glucose (**11**) [23,24], 1,6-di-*O*-galloyl-β-d-glucose (**12**) [25], 1,2,3-tri-*O*-galloyl-β-d-glucose (**13**) [5,26], 1,2,6-tri-*O*-galloyl-β-d-glucose (**14**) [5,26], 1,2,3,6-tetra-*O*-galloyl-β-d-glucose (**15**) [5,26], 1,2,3,4,6-penta-*O*-galloyl-β-d-glucose (**16**) [24,27], 1,6-di-*O*-galloyl-2-*O*-*p*-coumaroyl-β-d-glucose (**17**) [24], 1,6-di-*O*-galloyl-2-*O*-caffeoyl-β-d-glucose (**18**) [28], tellimagrandin I (**19**) [26,29], tellimagrandin II (**20**) [5,26], cornusiin A (**21**) [30], rugosin D (**22**) [31], and (7′*S*,8′*R*)-dihydrodehydrodiconiferyl alcohol-9′-*O*-β-d-glucose (**23**) [5] (Figure 1). The known compounds **2**–**23** were identified by direct HPLC comparison with authentic standards and/or by comparing their spectral data with those reported in the literature. To the best of our knowledge, vanillic acid (**4**), brevifolincarboxylic acid (**5**), urolithin A (**7**), urolithin M6 (**10**), and 1,6-di-*O*-galloyl-2-*O*-*p*-coumaroyl-β-d-glucose (**17**) were isolated for the first time from water chestnut in this study. 

**Figure 2:**
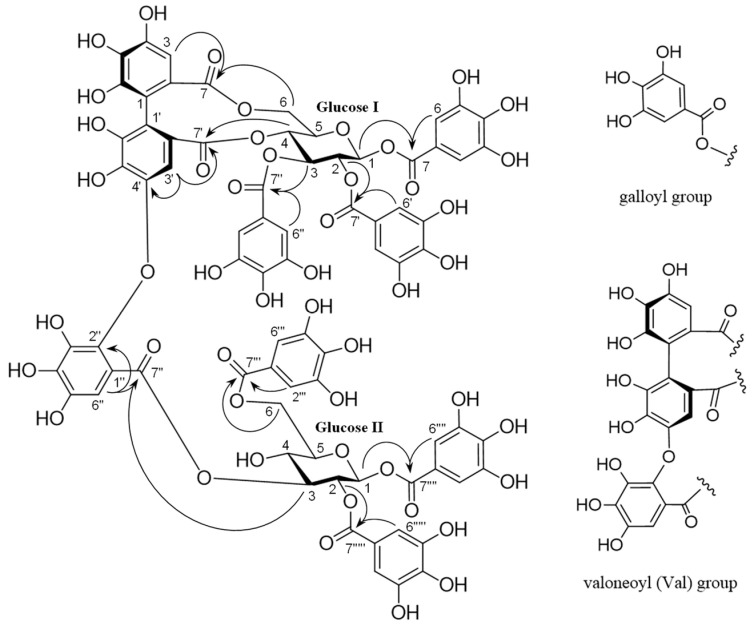
Key heteronuclear multiple-bond connectivity (HMBC) correlations (H→C) of **1**.

Trapadin A (**1**) was isolated as a light-brown amorphous powder. Its molecular formula was assigned as C_75_H_56_O_48_ based on high-resolution electrospray ionization mass spectrometry (HR-ESI-MS) results (*m*/*z* 1747.1838 [M + Na]^+^ calculated for C_75_H_56_O_48_ + Na: 1747.1833). The ultra-violet (UV) spectrum showed absorption maxima at 217 and 279 nm. Its ^1^H nuclear magnetic resonance (NMR) spectrum (500 MHz, in MeOH-*d*_4_) showed that it comprised six galloyl (Gal) groups (δ 7.10, 7.03, 7.01, 6.99, 6.91, 6.84 (each 2H, s)), a valoneoyl (Val) group (δ 6.95 (Val H-6″), 6.60 (Val H-3), 6.21 (Val H-3′)), and two sets of sugar proton signals (Table 1). The presence of two β-glucose units with the ^4^C_1_ conformation in **1** was indicated by two anomeric proton signals at δ 6.03 (d, *J* = 8.0 Hz) and δ 6.04 (d, *J* = 8.0 Hz) and the coupling pattern assigned using ^1^H–^1^H correlation spectroscopy (COSY). The presence of a free hydroxyl group at the C-4 of glucose II was also based on the appearance of H-1–H-3 and H-6 signals in a lower field (δ 6.04–4.37) than the H-4 signal (δ 3.65), as shown in Table 1. The ^13^C NMR spectrum (126 MHz, in MeOH-*d*_4_), exhibiting 12 carbon signals (δ 94.2, 93.8, 72.4, 72.7, 73.9, 76.7, 71.7, 69.7, 73.5, 76.9, 63.9, and 65.0) in the aliphatic region, assignable to two glucose units, also supported their existence (Table 1). The NMR data were similar to those of woodfordin A [32] and cornusiin G [33], which are dimeric tannins formed from units of tellimagrandin II and 1,2,3,6-tetra-*O*-galloyl-β-d-glucose. The linking position for each unit was determined using key heteronuclear multiple-bond connectivity (HMBC) correlations from glucose I H-4 (δ 5.09) and Val H-3′ (δ 6.21) to Val C-7′ (δ 169.0), from Val H-3′ (δ 6.21) to Val C-4′ (δ 147.9), from glucose I H-6 (δ 3.87, 5.38) and Val H-3 (δ 6.60) to Val C-7 (δ 169.4), from glucose II H-3 (δ 5.51) and Val H-6″ (δ 6.95) to Val C-7″ (δ 166.8), and from Val H-6″ (δ 6.95) to Val C-2″ (δ 137.1). The proton signals of the galloyl groups were correlated through each ester carbonyl carbon with the proton signals of each glucose, as shown in Figure 2; therefore, the galloyl groups were assigned to be at O-1, -2, and -3 on glucose I and at O-1, -2, and -6 on glucose II. The absolute configuration of the valoneoyl group in **1** was determined; a positive Cotton effect ([θ]_224_ +1.8 × 10^5^) in the short wavelength of the circular dichroism (CD) spectrum of **1** (in MeOH) indicated the (*S*)-configuration of the Val group [30,34]. Methylation of **1** followed by methanolysis yielded a methyl tri-*O*-methylgallate (**24**) and a trimethyl octa-*O*-methylvaloneate (**25**) (Figure 1). Furthermore, the (*S*)-configuration of the Val group of **1** was supported by a positive Cotton effect ([θ]_219_ + 0.5 × 10^5^) in the CD spectrum of **25**, similarly to that observed in **1** [35].

Two carbonyls of the hexahydroxydiphenoyl (HHDP) part of the Val group were located at O-4 and O-6 of the glucose I core in **1**, which indicated the existence of two regioisomers. Orientation of the Val group in **1** was found to be of the isorugosin type, similar to that in cornusiin G, based on a comparison of the ^1^H NMR signal (δ 6.60) for Val H-3 and the ^13^C NMR signal (δ 147.9) for Val C-4″ of **1** with those of the rugosin and isorugosin types [31,33,36,37]. The sugar unit obtained following acid hydrolysis of **1** was identified as D-glucose based on HPLC analysis of the derivatives prepared by the reaction with L-cysteine methyl ester and *o*-tolyl isothiocyanate according to a previously reported method [35]. Based on these data, the structure of trapadin A was established as **1**.

### 2.2. DPPH Radical Scavenging Activity

Several methods for evaluating antioxidant activity based on their principles and simplicity have been reported. Among these, the DPPH assay showed relatively high measurement accuracy for single compounds [38]. The DPPH method has extremely high reproducibility and is effective as a standard test method [39]. Therefore, in this study, the antioxidant activity was evaluated using the DPPH method.

DPPH radical scavenging activity assays were performed for compounds **1**–**23** (Table 2). The degree of activity was indicated by the half-maximal effective concentration (EC_50_) values. Compounds **4** and **7**–**9** were inactive (EC_50_: >100 μM), as evidenced by their antioxidant activity, and none of them had adjacent phenolic hydroxyl groups in its molecules. All the other compounds had adjacent phenolic hydroxyl groups in their molecules, and the greater the number, the stronger the activity. The EC_50_ values showed notable activity for hydrolyzable tannins. The EC_50_ values of hydrolyzable tannin monomers and dimers were between 5.06 and 12.5 µM and 3.48 and 3.91 µM, respectively, and the dimer with a higher number of phenolic hydroxyl groups in the molecule exhibited enhanced activity. Among these values, the EC_50_ value of trapadin A (**1**), the new compound, was 3.14 µM, demonstrating the highest activity. Therefore, we concluded that hydrolyzable tannins, especially dimers, contribute enormously to the antioxidant activity of water chestnuts. Therefore, further investigation of hydrolyzable tannin oligomers in water chestnuts is necessary.

## 3. Experimental Section

### 3.1. General

Optical rotations were measured using a JASCO-P-1020 digital polarimeter (JASCO Corporation, Tokyo, Japan). The UV spectra were recorded using a Shimadzu UVmini-1240 (Shimadzu Corporation, Kyoto, Japan). The HR-ESI-MS spectra were obtained using a micrOTOF-Q mass spectrometer (Bruker Daltonics, Billerica, MA, USA) with MeOH as the solvent. The NMR spectra were recorded using a Bruker AVANCE500 instrument (Bruker Biospin, Billerica, MA, USA; 500 and 126 MHz for ^1^H and ^13^C, respectively), and chemical shifts were expressed as parts per million (ppm) relative to those of the solvents (MeOH-*d*_4_ (δ_H_ 3.30; δ_C_ 49.0) and acetone-*d*_6_ (δ_H_ 2.04; δ_C_ 29.8)) on a tetramethylsilane scale. The standard pulse sequences programmed for the instrument (AVANCE500) were used for each 2D measurement (COSY, HSQC, and HMBC). The *J*_CH_ was set at 10 Hz for HMBC analysis. Column chromatography was performed using Diaion HP-20, MCI-gel CHP-20P (Mitsubishi Chemical Co., Tokyo, Japan), Chromatorex ODS (Fuji Silysia Chemical Ltd., Aichi, Japan), Sephadex LH-20 (Cytiva, Tokyo, Japan), and YMC GEL ODS (YMC Co. Ltd., Kyoto, Japan) columns. The reversed-phase (RP) HPLC conditions were as follows: Condition 1—column, YMC-pack ODS AQ-3C2 (5 µm, 150 × 2.0 mm i.d., YMC Co., Ltd., Kyoto, Japan); mobile phase, 10 mmol/L phosphate buffer–acetonitrile (9:1); column temperature, 40 °C; flow rate, 0.2 mL/min; detection wavelength, 280 nm. Condition 2—column, L-column ODS (5 µm, 150 × 2.1 mm i.d., Chemicals Evaluation and Research Institute, Tokyo, Japan); mobile phase, solvent A was 0.1% formic acid in water, and solvent B was 0.1% formic acid in acetonitrile (0–30 min, 0–50% B in A; 30–35 min, 50–85% B in A; 35–40 min, 85% B in A; 40–50 min, 85–100% B in A); injection volume, 3 µL; column temperature, 40 °C; flow rate, 0.3 mL/min; and detection wavelength, 200–400 nm. Condition 3—column, L-column ODS (5 µm, 150 × 2.1 mm i.d.); mobile phase, 0.1% formic acid in water–0.1% formic acid in acetonitrile (75:25); column temperature, 35 °C; flow rate, 0.3 mL/min; detection wavelength, 250 nm. The normal-phase (NP) HPLC condition was as follows: Condition 4—column, SILICA SG80 (5 µm, 150 × 2.0 mm i.d., Shiseido Co., Ltd., Tokyo, Japan); mobile phase, *n*-hexane–MeOH–tetrahydrofran–formic acid (55:33:11:1) containing oxalic acid (450 mg/L); column temperature, room temperature; flow rate, 0.3 mL/min; detection wavelength, 280 nm. Analytical and preparative thin-layer chromatography (TLC) was performed on TLC Silica gel 60F_254_ plates (Merck, Darmstadt, Germany).

### 3.2. Materials

The water chestnuts (lot. nos. 06047F209 and 06047D280, the fruit of *Trapa bispinosa*) used for the phytochemical investigation were purchased from Nakajima Shoyaku Ltd. (Kyoto, Japan). All other reagents used were of special or analytical grade.

### 3.3. Extraction and Isolation

Water chestnuts (625 g) were homogenized in 80% MeOH (MeOH–H_2_O (8:2), 6.4 L); the homogenate was filtered and concentrated to yield 20 g of residue. A portion (17 g) of the residue suspended in H_2_O was separated using column chromatography on a Diaion HP-20 column with an aqueous MeOH in a stepwise gradient mode as follows: 0:100→10:90→20:80→30:70→50:50→100:0.

A 10% MeOH eluate (700 mg) was separated using column chromatography over MCI-gel CHP-20P with aqueous MeOH to obtain brevifolincarboxylic acid (**5**, 4.7 mg), 1,2-di-*O*-galloyl-β-d-glucose (**11**, 5.3 mg), and 1,6-di-*O*-galloyl-β-d-glucose (**12**, 2.1 mg). A 30% MeOH eluate (393.7 mg) was separated using column chromatography over Sephadex LH-20 with EtOH and/or YMC GEL ODS with aqueous MeOH to obtain methyl gallate (**3**, 8.1 mg), 1,2,3-tri-*O*-galloyl-β-d-glucose (**13**, 10.5 mg), tellimagrandin I (**19**, 15.0 mg), and cornusiin A (**21**, 7.4 mg). A 50% MeOH eluate (700 mg) was separated using column chromatography over Sephadex LH-20 with EtOH and/or YMC GEL ODS and/or MCI-gel CHP-20P with aqueous MeOH to obtain gallic acid (**2**, 4.1 mg), vanillic acid (**4**, 3.2 mg), ellagic acid (**6**, 3.9 mg), 1,2,6-tri-*O*-galloyl-β-d-glucose (**14**, 41.0 mg), 1,2,3,6-tetra-*O*-galloyl-β-d-glucose (**15**, 146.2 mg), 1,2,3,4,6-penta-*O*-galloyl-β-d-glucose (**16**, 9.4 mg), tellimagrandin II (**20**, 104.6 mg), 1,6-di-*O*-galloyl-2-*O*-*p*-coumaroyl-β-d-glucose (**17**, 11.3 mg), 1,6-di-*O*-galloyl-2-*O*-caffeoyl-β-d-glucose (**18**, 1.8 mg), (7′*S*,8′*R*)-dihydrodehydrodiconiferyl alcohol-9′-*O*-β-d-glucose (**23**, 4.6 mg), rugosin D (**22**, 19.0 mg), and trapadin A (**1**, 26.5 mg). A MeOH eluate (800 mg) was separated using column chromatography over Sephadex LH-20 and/or MCI-gel CHP-20P with aqueous MeOH to obtain urolithin A (**7**, 3.8 mg), isourolithin A (**8**, 0.8 mg), urolithin B (**9**, 5.4 mg), and urolithin M6 (**10**, 1.1 mg). These compounds were identified following direct comparison with authentic standards or by comparing their spectral data with those reported in the literature. The physical and spectral data of the new compound **1** are as follows.

Trapadin A (**1**): A light-brown amorphous powder. HR-ESI-MS *m*/*z*: 1747.1838 ([M + Na]^+^, calcd. for C_75_H_56_O_48_ + Na: 1747.1833). UV λ_max_ (MeOH) nm (log ε): 217 (5.22), 279 (4.88). [α]_D_^22^ +14° (*c* = 0.2, MeOH). CD (MeOH) [α] (nm) +1.8 × 10^5^ (224), +0.4 × 10^5^ (238), −0.5 × 10^5^ (258), +0.6 × 10^5^ (280),^1^H-NMR (500 MHz, MeOH-*d*_4_) δ: 7.10, 7.03, 7.01, 6.99, 6.91, 6.84 (each 2H, s, galloyl-H), 6.95 (1H, s, Val H-6″), 6.60 (1H, s, Val H-3), 6.21 (1H, s, Val H-3′), glucose protons data are provided in Table 1. ^13^C-NMR (126 MHz, MeOH-*d*_4_) δ: 169.4 (Val C-7), 169.0 (Val C-7′), 168.4, 167.3, 167.2, 166.9, 166.4, 166.3 (galloyl C-7), 166.8 (Val C-7″), 147.9 (Val C-4′), 146.54, 146.48, 146.44, 146.37, 146.34, 146.02 (galloyl C-3 and C-5), 145.98 (Val C-4), 145.4, 145.1 (Val C-6, C-6′), 143.8 (Val C-5″), 141.2 (Val C-4″), 140.78 (2C), 140.66, 140.26, 140.23, 139.96, 139.92 (galloyl C-4, Val C-3″), 137.8 (Val C-5′), 137.4 (Val C-5), 137.1 (Val C-2″), 126.03, 126.01 (Val C-2, C-2′), 121.3, 120.6, 120.4, 120.3, 119.9, 119.7 (galloyl C-1), 117.9 (Val C-1′), 116.2 (Val C-1), 114.9 (Val C-1″), 110.69, 110.68, 110.62, 110.5 (3C), 110.4, 110.3 (galloyl C-2 and C-6, Val C-6″), 108.4 (Val C-3), 105.9 (Val C-3′), glucose carbon data are provided in Table 1.

### 3.4. Methylation of Compound ***1*** Followed by Methanolysis

A solution of compound **1** (4 mg) in EtOH (2 mL) was treated with (trimethylsilyl)diazomethane in hexane solution (2 mL) at room temperature overnight. After solvent removal, the residue was directly methanolyzed without further purification using 0.2% sodium methoxide in MeOH (2 mL) at room temperature for 12 h. After acidification with a few drops of 10% hydrochloric acid, the reaction mixture was evaporated, and the residue was purified using preparative TLC (*n*-hexane–acetone (1:1)) to yield methyl tri-*O*-methylgallate (**24**, 1.7 mg, HR-ESI-MS *m*/*z*: 249.0752 (M + Na)^+^, calcd. for C_11_H_14_O_5_ + Na: 249.0733) and trimethyl octa-*O*-methylvaloneate (**25**; 1.0 mg, HR-ESI-MS *m*/*z*: 683.1958 (M + Na)^+^, calcd. for C_32_H_36_O_15_ + Na: 638.1946, CD (MeOH) [θ] (nm): +0.5 × 10^5^ (219), −0.3 × 10^5^ (249). ^1^H-NMR (in acetone-*d*_6_) δ: 7.38, 7.31, 6.90 (each 1H, s), 4.02, 3.95, 3.94, 3.93, 3.86, 3.76, 3.75, 3.62, 3.55, 3.54, 3.41 (each 3H, s)), which were identified by comparison with an authentic sample.

### 3.5. Determination of the Sugar Configuration of Compound ***1***

The sugar configuration was determined using a previously described method [35]. Compound **1** (1.0 mg) was hydrolyzed by heating in 1 mol/L hydrochloric acid (0.2 mL) and neutralized using Amberlite IRA400 (Organo Corporation, Tokyo, Japan). After evaporation, the residue was dissolved in pyridine (0.2 mL) containing L-cysteine methyl ester hydrochloride (1.0 mg) and heated at 60 °C for 1 h. *o*-Tolyl isothiocyanate (1.0 mg) in pyridine (0.2 mL) was then added to each mixture, followed by direct analysis using RP-HPLC (Condition 3). The peak from compound **1** coincided with that of the derivative similarly prepared from an authentic D-glucose sample.

### 3.6. DPPH Radical Scavenging Activities of Compounds ***1***–***23***

The DPPH radical scavenging activity of each compound was determined using the DPPH Antioxidant Assay Kit (Dojin Laboratories, Kumamoto, Japan) following the manufacturer’s instructions [39,40]. The sample solution, assay buffer, and DPPH working solution were mixed in 96-well plates and incubated in the dark at 25 °C for 30 min. The absorbance was measured at 517 nm using an Infinite F200 microplate reader (Tecan Group Ltd., Mannedorf, Switzerland). The EC_50_ was determined via regression line analysis, and Trolox was used as the positive control. All experiments were performed in triplicate.

## 4. Conclusions

In conclusion, we successfully isolated a new hydrolyzable tannin dimer, trapadin A (**1**), from water chestnut, along with 22 known compounds. Through the application of spectroscopic and chemical methods, the structure of trapadin A (**1**) was elucidated, revealing a hydrolyzable tannin dimer formed from units of tellimagrandin II and 1,2,3,6-tetra-*O*-galloyl-β-d-glucose. Furthermore, the determined EC_50_ values in the DPPH radical scavenging assay revealed substantial activity for hydrolyzable tannins, with trapadin A (**1**), the new hydrolyzable tannin dimer, exhibiting the highest activity value. The findings of this study suggest that the hydrolyzable tannin dimers are responsible for the antioxidant activity of the water chestnut.

## Figures and Tables

**Table 1:** ^1^H and ^13^C NMR data for glucose residues of compound **1** measured in MeOH-*d*_4_.

Position	δ_H_ (*J* in Hz)	δ_C_
Glucose I		
H-1	6.03 (d, *J* = 8.0)	94.2
H-2	5.48 (dd, *J* = 8.0, 9.5)	72.4
H-3	5.63 (t, *J* = 9.5)	73.9
H-4	5.09 (t, *J* = 9.5)	71.7
H-5	4.41 (m)	73.5
H-6	5.38 (m), 3.87 (br d, *J* = 12.5)	63.9
Glucose II		
H-1	6.04 (d, *J* = 8.0)	93.8
H-2	5.39 (dd, *J* = 8.0, 9.5)	72.7
H-3	5.51 (t, *J* = 9.5)	76.7
H-4	3.65 (t, *J* = 9.5)	69.7
H-5	3.90 (m)	76.9
H-6	4.68 (dd, *J* = 2.0, 12.5), 4.37 (dd, *J* = 7.0, 12.5)	65.0

**Table 2:** DPPH radical scavenging activities of compounds **1**–**23**.

Compounds	EC_50_ (µM)
Trapadin A (**1**)	3.14
Gallic acid (**2**)	10.8
Methyl gallate (**3**)	21.1
Vanillic acid (**4**)	>100
Brevifolincarboxylic acid (**5**)	31.5
Ellagic acid (**6**)	15.7
Urolithin A (**7**)	>100
Isourolithin A (**8**)	>100
Urolithin B (**9**)	>100
Urolithin M6 (**10**)	23.3
1,2-Di-*O*-galloyl-β-d-glucose (**11**)	12.5
1,6-Di-*O*-galloyl-β-d-glucose (**12**)	12.3
1,2,3-Tri-*O*-galloyl-β-d-glucose (**13**)	7.63
1,2,6-Tri-*O*-galloyl-β-d-glucose (**14**)	7.79
1,2,3,6-Tetra-*O*-galloyl-β-d-glucose (**15**)	6.55
1,2,3,4,6-Penta-*O*-galloyl-β-d-glucose (**16**)	5.06
1,6-Di-*O*-galloyl-2-*O*-*p*-coumaroyl-β-d-glucose (**17**)	12.0
1,6-Di-*O*-galloyl-2-*O*-caffeoyl-β-d-glucose (**18**)	9.53
Tellimagrandin I (**19**)	5.75
Tellimagrandin II (**20**)	5.91
Cornusiin A (**21**)	3.48
Rugosin D (**22**)	3.91
(7′*S*,8′*R*)-Dihydrodehydrodiconiferyl alcohol-9′-*O*-β-d-glucose (**23**)	>100
Trolox	26.1

## Data Availability

Data are contained within the article and Supplementary Materials.

## Supplementary Materials

The following supporting information can be downloaded at: https://www.mdpi.com/article/10.3390/molecules28186563/s1, Figure S1: ^1^H-NMR spectrum of compound **1**; Figure S2: ^1^H-^1^H COSY spectrum of compound **1**; Figure S3: ^13^C-NMR spectrum of compound **1**; Figure S4: HSQC spectrum of compound **1**; Figure S5: HMBC spectrum of compound **1**; Figure S6: Selected HMBC correlations of compound **1**; Table S1: NMR data of woodfordin A and cornusiin G, which are dimeric hydrolyzable tannins formed from units of tellimagrandin II and 1,2,3,6-tetra-*O*-galloyl-β-d-glucose; spectral data of compounds **2**–**23**.

## Sample Availability

Not available.
