# Supplementary material for: Identification of Antioxidative Hydrolyzable Tannins in Water Chestnut
Source: Molecules. 2023 Sep 11;28(18):6563. doi: 10.3390/molecules28186563 (PMC10534751; doi:10.3390/molecules28186563)
Supplement: Supplementary file 1 [file molecules-28-06563-s001.zip › molecules-2578269-supplementary.pdf]

Supplementary Materials

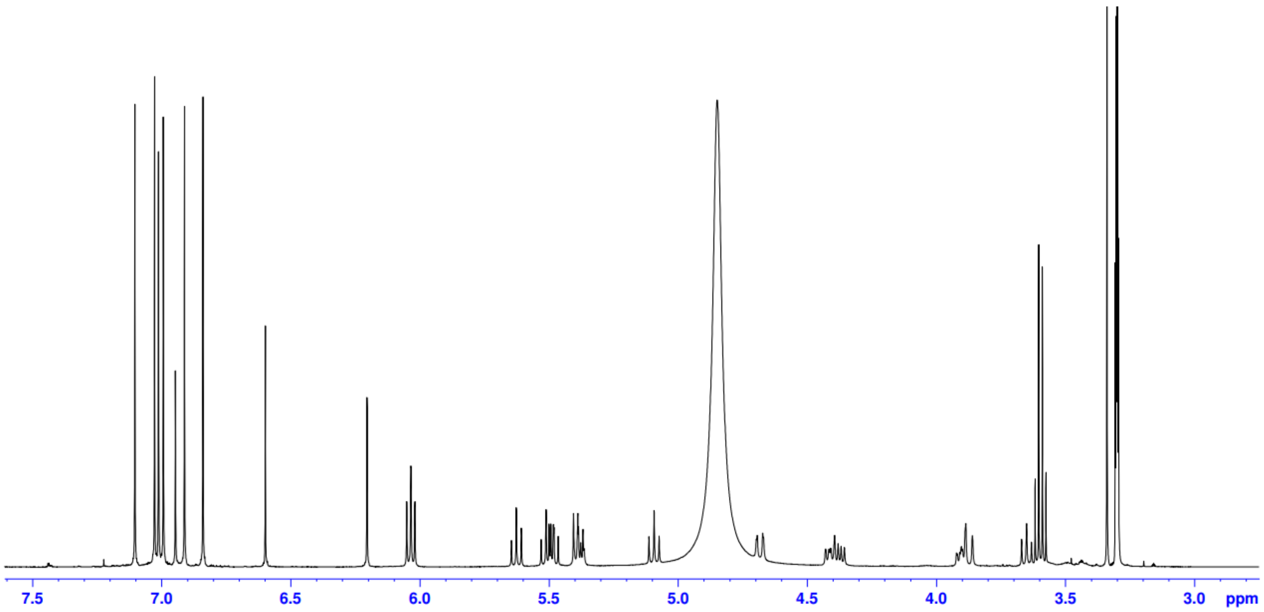

Figure S1. ^{1}H-NMR spectrum of compound **1**.

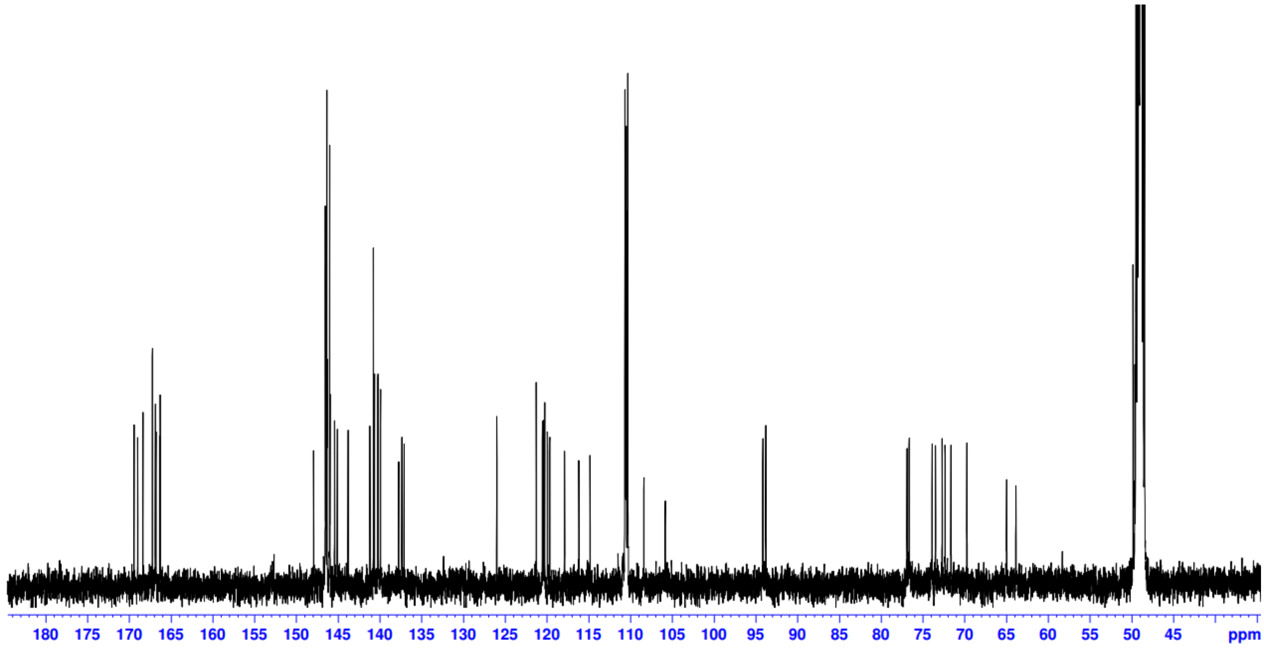

Figure S2. ^{13}C-NMR spectrum of compound **1**.

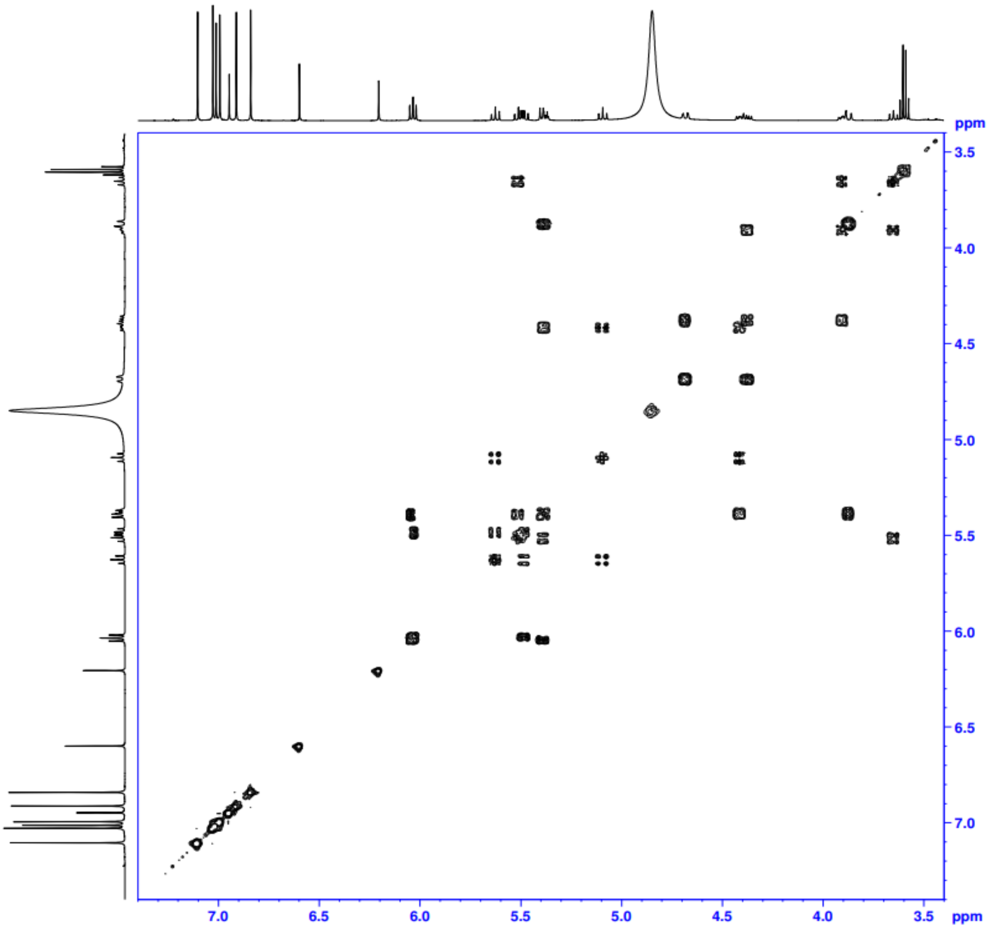

Figure S3. ^{1}H-^{1}H COSY spectrum of compound **1**.

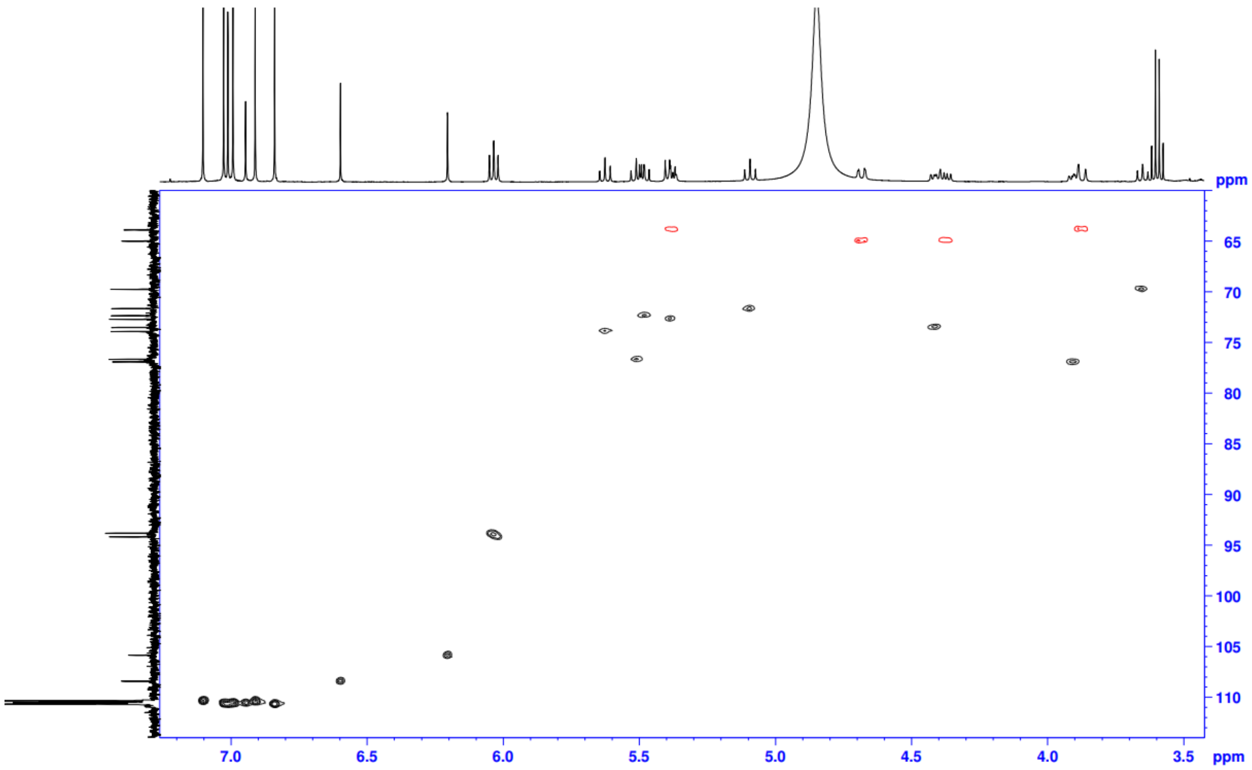

Figure S4. HSQC spectrum of compound **1**.

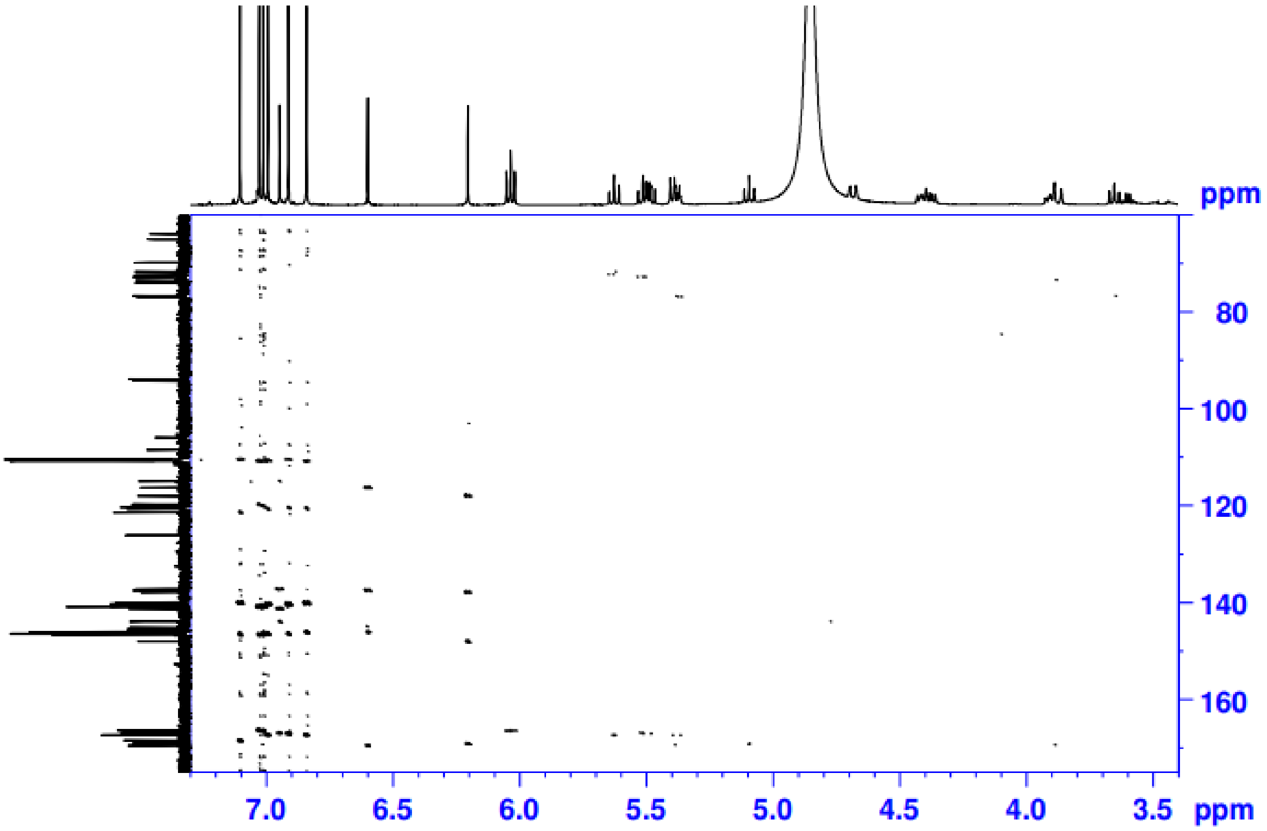

Figure S5. HMBC spectrum of compound **1.**

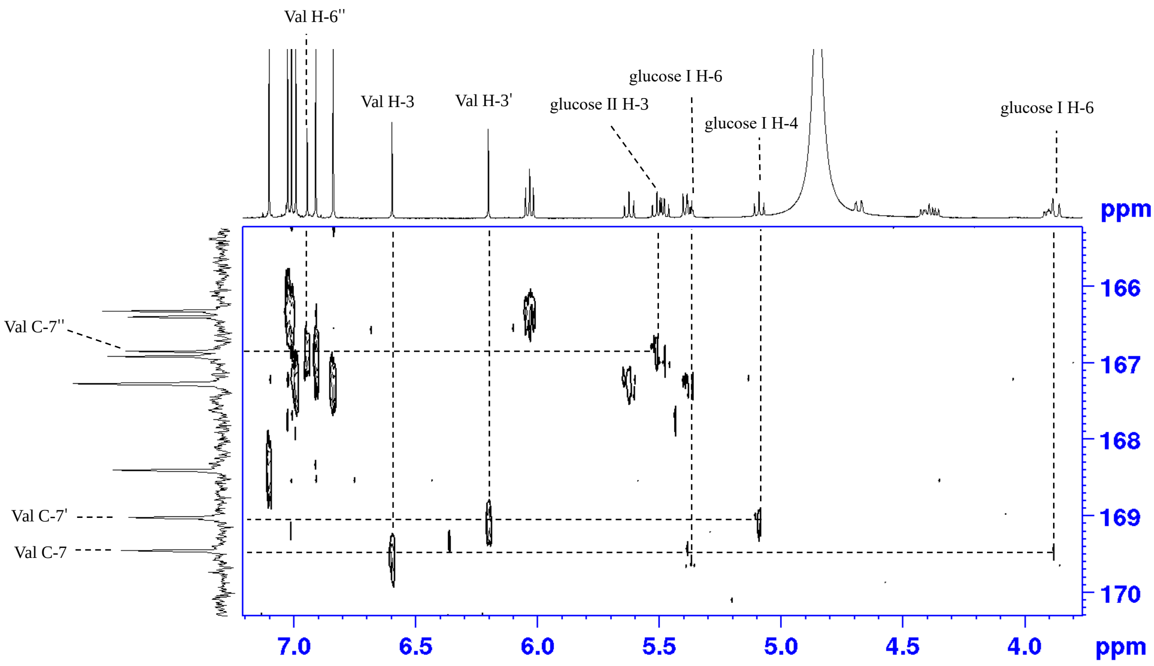

Figure S6. Selected HMBC correlations of compound **1**.

Table S1. NMR data of woodfordin A and cornusiin G which are dimeric hydrolyzable tannins formed from units of tellimagrandin II and 1,2,3,6-tetra-*O*-galloyl-β-D-glucose (Acetone-d_6 + D_2O).

	Woodfordin A[1)]		Cornusiin G[2)]	
Glucose I	^{1}H	^{13}C	^{1}H	^{13}C
1	6.15 (d, *J* = 8.5)	93.5	6.16 (d, *J* = 8)	93.6
2	5.57 (dd, *J* = 8.5, 10)	71.8	5.54 (dd, *J* = 8, 9.5)	71.7
3	5.81 (t, *J* = 10)	73.1	5.60 (t, *J* = 10)	73.1
4	5.16 (t, *J* = 10)	70.7	5.08 (t, *J* = 10)	71.0
5	4.49 (dd, *J* = 6.5, 10)	72.7	4.49 (dd, *J* = 6.5, 13)	72.9
6	5.23 (dd, *J* = 6.5, 13)	63.2	5.39 (dd, *J* = 6.5, 13)	63.2
	3.78 (d, *J* = 13)		3.87 (d, *J* = 13)	
Glucose II				
1'	6.08 (d, *J* = 8.5)	93.1	6.05 (d, *J* = 8)	93.3
2'	5.43 (d, *J* = 8.5, 10)	72.0	5.40 (dd, *J* = 8, 9.5)	72.1
3'	5.60 (t, *J* =1 0)	75.5	5.58 (t, *J* = 10)	75.8
4'	3.68 (t, *J* = 10)	69.4	3.80 (t, *J* = 9.5)	69.4
5'	4.06 (m)	76.2	4.06 (ddd, *J* = 2, 6.5, 10)	76.2
6'	4.68 (dd, *J* = 2, 12.5)	64.5	4.70 (dd, *J* = 2, 12)	64.5
	4.43 (dd, *J* = 6.5, 12.5)		4.39 (dd, *J* = 6.5, 12.5)	
Galloyl-H	7.15, 7.08, 7.06 (each 2H, s), 6.99 (6H, s)		7.14, 7.09, 7.06, 7.03, 6.98, 6.89 (each 2H, s)	
Valloneoyl-H	7.04, 6.49, 6.19 (each 1H, s)		7.07, 6.62, 6.19 (each 1H, s)	

1) Yoshida T. et al., *Heterocycles* **1989**, *29*, 2267-2271.
2) Hatano, T. et al., *Phytochemistry* **1990**, *29*, 2975-2978.

Spectral data of Compounds 2-23

Gallic acid (**2**): A pale yellow amorphous powder. HR-ESI-MS *m/z*: 169.0141 ([M-H]$^-$, Calcd. for $C_7H_6O_5$-H: 169.0142). ^{1}H-NMR (500 MHz, acetone-d_6 + D_2O) δ: 7.08 (2H, s, H-2, 6). ^{13}C-NMR (126 MHz, acetone-d_6 + D_2O) δ: 168.6 (C-7), 145.9 (C-3, 5), 138.7 (C-4), 122.1 (C-1), 109.9 (C-2, 6).

Methyl gallate (**3**): A pale yellow amorphous powder. HR-ESI-MS *m/z*: 183.0287 ([M-H]$^-$, Calcd. for $C_8H_8O_5$-H: 183.0299). ^{1}H-NMR (500 MHz, acetone-d_6 + D_2O) δ: 7.06 (2H, s, H-2, 6), 3.75 (3H, s, H-8). ^{13}C-NMR (126 MHz, acetone-d_6 + D_2O) δ: 167.6 (C-7), 146.0 (C-3, 5), 138.8 (C-4), 121.3 (C-1), 109.6 (C-2, 6), 51.9 (C-8).

Vanillic acid (**4**): A pale yellow amorphous powder. HR-ESI-MS *m/z*: 167.0344 ([M-H]$^-$, Calcd. for $C_8H_8O_4$-H: 167.0350). ^{1}H-NMR (500 MHz, MeOH-d_4) δ: 7.55-7.53 (2H, m, H-2, 6), 6.82 (1H, d, *J* = 8.0 Hz, H-5), 3.88 (3H, s, 3-OCH_3).

Brevifolincarboxylic acid (**5**): A yellow crystalline powder. HR-ESI-MS *m/z*: 291.0137 ([M-H]$^-$, Calcd. for $C_{13}H_8O_8$-H: 291.0146). ^{1}H-NMR (500 MHz, acetone-d_6 + D_2O) δ: 7.34 (1H, s, H-3'), 4.44 (1H, d, *J* = 6.5 Hz, H-4), 2.99 (1H, d, *J* = 18.5 Hz, H-5), 2.66 (1H, dd, *J* = 6.5, 18.5 Hz, H-5).

Ellagic acid (**6**): A pale yellow amorphous powder. HR-ESI-MS *m/z*: 301.0003 ([M-H]$^-$, Calcd. for $C_{14}H_6O_8$-H: 300.9990). ^{1}H-NMR (500 MHz, acetone-d_6 + D_2O) δ: 7.53 (2H, s, H-5, 5').

Urolithin A (**7**): An off-white amorphous powder. HR-ESI-MS *m/z*: 227.0354 ([M-H]$^-$, Calcd. for $C_{13}H_8O_4$-H: 227.0350). ^{1}H-NMR (500 MHz, MeOH-d_4) δ: 8.02 (1H, d, *J* = 8.5 Hz, H-10), 7.93 (1H, d, *J* = 9.0, H-1), 7.60 (1H, d, *J* = 3.0 Hz, H-7), 7.31 (1H, dd, *J* = 3.0, 8.5 Hz, H-9), 6.81 (1H, dd, *J* = 2.5, 9.0 Hz, H-2), 6.72 (1H, d, *J* = 2.5 Hz, H-4).

Isourolithin A (**8**): An off-white amorphous powder. HR-ESI-MS *m/z*: 227.0380 ([M-H]$^-$, Calcd. for $C_{13}H_8O_4$-H: 227.0350). ^{1}H-NMR (500 MHz, MeOH-d_4) δ: 8.12 (1H, d, *J* = 8.5 Hz, H-7), 7.92 (1H, d, *J* = 8.5, H-1), 7.40 (1H, d, *J* = 2.5 Hz, H-10), 6.94 (1H, dd, *J* = 2.5, 8.5 Hz, H-8), 6.83 (1H, dd, *J* = 2.5, 8.5 Hz, H-2), 6.71 (1H, d, *J* = 2.5 Hz, H-4).

Urolithin B (**9**): An off-white amorphous powder. HR-ESI-MS *m/z*: 211.0422 ([M-H]$^-$, Calcd. for $C_{13}H_8O_3$-H: 211.0401).^{1}H-NMR (500 MHz, MeOH-d_4) δ: 8.25 (1H, dd, *J* = 1.0, 8.0 Hz, H-7), 8.15 (1H, brd, *J* = 8.0, H-10), 8.05 (1H, d, *J* = 9.0 Hz, H-1), 7.83 (1H, ddd, *J* = 1.0, 8.0, 8.5 Hz, H-9), 7.51 (1H, ddd, *J* = 1.0, 8.0, 8.5 Hz, H-8), 6.85 (1H, dd, *J* = 2.5, 9.0 Hz, H-2), 6.74 (1H, d, *J* = 2.5 Hz, H-4). ^{13}C-NMR (126 MHz, MeOH-d_4) δ: 163.4 (C-6), 161.4 (C-3), 153.9 (C-4a), 137.1 (C-10a), 136.4 (C-9), 131.1 (C-7), 128.6 (C-8), 125.5 (C-1), 122.5 (C-10), 120.6 (C-6a), 114.4 (C-2), 111.3 (C-10b), 104.2 (C-4).

Urolithin M6 (**10**): An off-white amorphous powder. HR-ESI-MS *m/z*: 259.0278 ([M-H]$^-$, Calcd. for $C_{13}H_8O_6$-H: 259.0248). ^{1}H-NMR (500 MHz, MeOH-d_4) δ: 7.08, 7.06 (each 2H, s, Galloyl-H), 5.17 (1H, dd, *J* = 8.5, 9.5 Hz, H-7), 6.75 (1H, dd, *J* = 2.5, 9.0 Hz, H-2), 6.69 (1H, d, *J* = 2.5 Hz, H-4). ^{13}C-NMR (126 MHz, MeOH-d_4) δ: 164.3 (C-6), 158.9 (C-3), 152.4 (C-4a), 146.5 (C-8), 143.8 (C-10), 142.0 (C-9), 129.8 (C-1), 118.2 (C-10a), 113.2 (C-2), 112.21 (C-10b), 112.17 (C-6a), 108.1 (C-7), 103.7 (C-4).

1,2-Di-*O*-galloyl-β-D-glucose (**11**): A pale yellow amorphous powder. HR-ESI-MS *m/z*: 483.0787 ([M-H]$^-$, Calcd. for $C_{20}H_{20}O_{14}$-H: 483.0780). ^{1}H-NMR (500 MHz, acetone-d_6 + D_2O) δ: 7.08, 7.06 (each 2H, s, Galloyl-H), 5.86 (1H, d, *J* = 8.0 Hz, glucose H-1), 5.17 (1H, dd, *J* = 8.5, 9.5 Hz, glucose H-2), 3.91-3.85 (3H, m, glucose H-3, 6), 3.72 (1H, m, glucose H-5), 3.59 (1H, m, glucose H-4).

1,6-Di-*O*-galloyl-β-D-glucose (**12**): A pale yellow amorphous powder. HR-ESI-MS *m/z*: 483.0793 ([M-H]$^-$, Calcd. for $C_{20}H_{20}O_{14}$-H: 483.0780). ^{1}H-NMR (500 MHz, acetone-d_6 + D_2O) δ: 7.15, 7.11 (each 2H, s, Galloyl-H), 5.70 (1H, d, *J* = 8.0 Hz, glucose H-1), 4.56 (1H, dd, *J* = 2.0, 12.0 Hz, glucose H-6), 4.35 (1H, dd, *J* = 5.5, 12.0 Hz, glucose H-6), 3.79 (1H, m, glucose H-5), 3.65-3.53 (3H, m, glucose H-2,3,4). ^{13}C-NMR (126 MHz, acetone-d_6 + D_2O) δ: 166.9, 165.8 (Galloyl C-7, 7'), 145.9 (2C, Galloyl C-3, 3', 5, 5'), 139.4, 138.8 (Galloyl C-4, 4'), 121.3, 120.5 (Galloyl C-1, 1'), 110.1, 109.9 (Galloyl C-2, 2', 6, 6'), 95.5 (glucose C-1), 77.5 (glucose C-3), 75.8 (glucose C-5), 73.5 (glucose C-2), 70.7 (glucose C-4), 64.2 (glucose C-6).

1,2,3-Tri-*O*-galloyl-β-D-glucose (**13**): A pale yellow amorphous powder. HR-ESI-MS *m/z*: 635.0905 ([M-H]$^-$, Calcd. for $C_{27}H_{24}O_{18}$-H: 635.0890). ^{1}H-NMR (500 MHz, acetone-d_6 + D_2O) δ: 7.07, 7.06, 6.98 (each 2H, s, Galloyl-H), 6.05 (1H, d, *J* = 8.5 Hz, glucose H-1), 5.59 (1H, t, *J* = 9.5 Hz, glucose H-3), 5.39 (1H, dd, *J* = 8.5, 9.5 Hz, glucose H-2), 3.96 (1H, t, *J* = 9.5 Hz, glucose H-4), 3.91 (1H, br d, *J* = 9.5 Hz, glucose H-3), 3.82-3.77 (2H, m, glucose H-5, 6). ^{13}C-NMR (126 MHz, acetone-d_6 + D_2O) δ: 166.6, 166.3, 165.3 (Galloyl C-7, 7', 7''), 146.0, 145.9 (Galloyl C-3, 3', 3'', 5, 5', 5''), 139.7, 139.3, 139.0 (Galloyl C-4, 4', 4''), 121.0, 120.3, 119.8 (Galloyl C-1, 1', 1''), 110.1, 109.9 (2C) (Galloyl C-2, 2', 2'', 6, 6', 6''), 93.4 (glucose C-1), 78.5 (glucose C-5), 76.0 (glucose C-3), 71.9 (glucose C-2), 68.9 (glucose C-4), 61.6 (glucose C-6).

1,2,6-Tri-*O*-galloyl-β-D-glucose (**14**): A pale yellow amorphous powder. HR-ESI-MS *m/z*: 635.0880 ([M-H]$^-$, Calcd. for $C_{27}H_{24}O_{18}$-H: 635.0890). ^{1}H-NMR (500 MHz, acetone-d_6 + D_2O) δ: 7.15, 7.11, 7.07 (each 2H, s,

Galloyl-H), 5.96 (1H, d, *J* = 8.0 Hz, glucose H-1), 5.26 (1H, dd, *J* = 8.0, 9.5 Hz, glucose H-2), 4.62 (1H, dd, *J* = 2.0, 12.0 Hz, glucose H-6), 4.46 (1H, dd, *J* = 5.0, 12.0 Hz, glucose H-6), 4.01 (1H, t, *J* = 9.5 Hz, glucose H-3), 3.95 (1H, m, glucose H-5), 3.77 (1H, t, *J* = 9.5 Hz, glucose H-4). ^{13}C-NMR (126 MHz, acetone-d_6 + D_2O) δ: 166.9, 166.5, 165.3 (Galloyl C-7, 7', 7''), 145.9, 145.8 (Galloyl C-3, 3', 3'', 5, 5', 5''), 139.5, 139.0, 138.8 (Galloyl C-4, 4', 4''), 121.2, 121.0, 119.8 (Galloyl C-1, 1', 1''), 110.7, 110.0, 109.8 (Galloyl C-2, 2', 2'', 6, 6', 6''), 93.5 (glucose C-1), 75.9 (glucose C-5), 75.2 (glucose C-3), 73.8 (glucose C-2), 71.0 (glucose C-4), 64.0 (glucose C-6).

1,2,3,6-Tetra-*O*-galloyl-β-D-glucose (**15**): A pale yellow amorphous powder. HR-ESI-MS *m/z*: 787.1028 ([M-H]$^-$, Calcd. for $C_{34}H_{28}O_{22}$-H: 787.1000). ^{1}H-NMR (500 MHz, acetone-d_6 + D_2O) δ: 7.16, 7.09, 7.08, 7.01 (each 2H, s, Galloyl-H), 6.14 (1H, d, *J* = 8.0 Hz, glucose H-1), 5.68 (1H, dd, *J* = 9.0, 10.0 Hz, glucose H-3), 5.48 (1H, dd, *J* = 8.0, 10.0 Hz, glucose H-2), 4.67 (1H, dd, *J* = 2.0, 12.0 Hz, glucose H-6), 4.51 (1H, dd, *J* = 5.0, 12.5 Hz, glucose H-6), 4.16 (1H, m, glucose H-5), 4.08 (1H, t, *J* = 9.0 Hz, glucose H-4). ^{13}C-NMR (126 MHz, acetone-d_6 + D_2O) δ: 166.9, 166.6, 165.3, 165.3 (Galloyl C-7, 7', 7'', 7'''), 146.0, 145.9, 145.8 (Galloyl C-3, 3', 3'', 3''', 5, 5', 5'', 5'''), 139.7, 139.3, 139.0, 138.9 (Galloyl C-4, 4', 4'', 4'''), 121.1, 121.8, 120.1, 119.6 (Galloyl C-1, 1', 1'', 1'''), 110.1, 110.0, 109.9, 109.8 (Galloyl C-2, 2', 2'', 2''', 6, 6', 6'', 6'''), 93.4 (glucose C-1), 75.9 (glucose C-5), 75.7 (glucose C-3), 71.8 (glucose C-2), 69.4 (glucose C-4), 63.8 (glucose C-6).

1,2,3,4,6-Penta-*O*-galloyl-β-D-glucose (**16**): A pale yellow amorphous powder. HR-ESI-MS *m/z*: 939.1137 ([M-H]$^-$, Calcd. for $C_{41}H_{32}O_{26}$-H: 939.1109). ^{1}H-NMR (500 MHz, MeOH-d_4) δ: 7.11, 7.05, 6.98, 6.95, 6.90 (each 2H, s, Galloyl-H), 6.23 (1H, d, *J* = 8.0 Hz, glucose H-1), 5.90 (1H, t, *J* = 10.0 Hz, glucose H-3), 5.60 (1H, t, *J* = 10.0 Hz, glucose H-4), 5.58 (1H, dd, *J* = 8.0, 10.0 Hz, glucose H-2), 4.51 (1H, br d, *J* = 10.5 Hz, glucose H-6), 4.42-4.36 (2H, m, glucose H-5, 6).

1,6-Di-*O*-galloyl-2-*O*-*p*-coumaroyl-β-D-glucose (**17**): A pale yellow amorphous powder. HR-ESI-MS *m/z*: 629.1150 ([M-H]$^-$, Calcd. for $C_{29}H_{26}O_{16}$-H: 629.1148). ^{1}H-NMR (500 MHz, acetone-d_6 + D_2O) δ: 7.63 (1H, d, *J* = 16.0 Hz, H-7''), 7.45 (2H, d, *J* = 8.5 Hz, H-2'', 6''), 7.10 (2H, s, H-2''', 6'''), 7.05 (2H, s, H-2', 6'), 6.81 (1H, d, *J* = 8.5 Hz, H-3'', 5''), 6.30 (1H, d, *J* = 16.0 Hz, H-8''), 5.83 (1H, d, *J* = 8.0 Hz, glucose H-1), 5.16 (1H, t, *J* = 8.0 Hz, glucose H-2), 4.59 (1H, br d, *J* = 12.0 Hz, glucose H-6), 4.37 (1H, dd, *J* = 5.0, 12.0 Hz, glucose H-6), 3.92-3.88 (2H, m, glucose H-3, 5), 3.69 (1H, t, *J* = 9.5 Hz, glucose H-4). ^{13}C-NMR (126 MHz, acetone-d_6 + D_2O) δ: 167.6 (C-9''), 167.1 (C-7'''), 165.6 (C-7'), 160.8 (C-4''), 146.7 (C-7''), 146.0 (C-3', 5'), 145.9 (C-3''', 5'''), 139.7 (C-4'), 138.9 (C-4'''), 131.0 (C-2'', 6''), 126.2 (C-1''), 120.9 (C-1'''), 119.5 (C-1'), 116.5 (C-3'', 5''), 114.3 (C-8''), 110.0 (C-2', 6'), 109.7 (C-2''', 6'''), 93.5 (glucose C-1), 75.8 (glucose C-5), 74.8 (glucose C-3), 73.4 (glucose C-2), 70.9 (glucose C-4), 64.0 (glucose C-6).

1,6-Di-*O*-galloyl-2-*O*-caffeoyl-β-D-glucose (**18**): A pale yellow amorphous powder. HR-ESI-MS *m/z*: 645.1126 ([M-H]$^-$, Calcd. for $C_{29}H_{26}O_{17}$-H: 645.1097). ^{1}H-NMR (500 MHz, acetone-d_6 + D_2O) δ: 7.56 (1H, d, *J* = 16.0 Hz, H-7''), 7.10 (2H, s, H-2''', 6'''), 7.08 (1H, d, *J* = 2.0 Hz, H-2''), 7.04 (2H, s, H-2', 6'), 6.93 (1H, dd, *J* = 2.0, 8.0 Hz, H-6''), 6.79 (1H, d, *J* = 8.0 Hz, H-5''), 6.24 (1H, d, *J* = 16.0 Hz, H-8''), 5.82 (1H, d, *J* = 8.0 Hz, glucose H-1), 5.15 (1H, dd, *J* = 8.0, 10.0 Hz, glucose H-2), 4.58 (1H, dd, *J* = 2.0, 12.0 Hz, glucose H-6), 4.36 (1H, dd, *J* = 5.5, 12.0 Hz, glucose H-6), 3.90-3.87 (2H, m, glucose H-3, 5), 3.68 (1H, t, *J* = 10.0 Hz, glucose H-4). ^{13}C-NMR (126 MHz, acetone-d_6 + D_2O) δ: 167.5 (C-9''), 167.1 (C-7'''), 165.6 (C-7'), 149.1 (C-4''), 147.0 (C-3''), 146.2 (C-7''), 146.0 (C-3'), 145.9 (C-3'''), 139.7 (C-4'), 139.0 (C-4'''), 126.9 (C-1''), 122.7(C-6''), 121.0 (C-1'''), 119.6 (C-1'), 116.2 (C-5''), 114.9 (C-2''), 114.3 (C-8''), 110.0 (C-2', 6'), 109.8 (C-2''', 6'''), 93.5 (glucose C-1), 75.9 (glucose C-5), 74.9 (glucose C-3), 73.5 (glucose C-2), 70.9 (glucose C-4), 64.0 (glucose C-6).

Tellimagrandin I (**19**): A pale yellow amorphous powder. HR-ESI-MS *m/z*: 785.0822 ([M-H]$^-$, Calcd. for $C_{34}H_{26}O_{22}$-H: 785.0843). ^{1}H-NMR (500 MHz, acetone-d_6 + D_2O) δ: 7.01, 7.00 (2H in total, s, Galloyl-H), 6.95, 6.90 (2H in total, s, Galloyl-H), 6.61, 6.60 (1H in total, s, HHDP-H), 6.45, 6.43 (1H in total, s, HHDP-H), 5.82 (t, *J* = 10.0 Hz, *α*-glucose H-3), 5.55 (t, *J* = 9.5 Hz, β−glucose H-3), 5.50 (d, *J* = 3.5 Hz, *α*-glucose H-1), 5.25-5.19 (m, *α*-glucose H-6, β-glucose H-2, 6), 5.10-5.04 (m, *α*-glucose H-2, 4, β-glucose H-4), 5.03 (d, *J* = 8.0 Hz, β-glucose H-1), 4.62 (ddd, *J* = 1.5, 6.5, 10.0 Hz, *α*-glucose H-5), 4.21 (br dd, *J* = 5.5, 10.0 Hz, β-glucose H-5), 3.81 (dd, *J* = 1.5, 13.0 Hz, β-glucose H-6), 3.74 (dd, *J* = 1.5, 13.0 Hz, *α*-glucose H-6).

Tellimagrandin II (**20**): A pale yellow amorphous powder. HR-ESI-MS *m/z*: 937.0928 ([M-H]$^-$, Calcd. for $C_{41}H_{30}O_{26}$-H: 937.0953). ^{1}H-NMR (500 MHz, acetone-d_6 + D_2O) δ: 7.08, 6.99, 6.95 (each 2H, s, Galloyl-H), 6.63, 6.48 (each 1H, s, HHDP H-2, 2'), 6.16 (1H, d, *J* = 8.0 Hz, glucose H-1), 5.80 (1H, t, *J* = 10.0 Hz, glucose H-3), 5.59 (1H, dd, *J* = 8.5, 9.5 Hz, glucose H-2), 5.31 (1H, dd, *J* = 6.5, 13.0 Hz, glucose H-6), 5.19 (1H, t, *J* = 10.0 Hz, glucose H-4), 4.53 (1H, m, glucose H-5), 3.87 (1H, br d, *J* = 13.0 Hz, glucose H-6). ^{13}C-NMR (126 MHz, acetone-d_6 + D_2O) δ: 168.3, 167.8, 166.7, 166.1, 165.2 (Galloyl C-7, 7', 7'', HHDP C-7, 7'), 146.1, 145.9, 145.7 (2C, Galloyl C-3, 3', 3'', 5, 5', 5''), 145.17, 145.14, 144.3 (2C) (HHDP C-4, 4', 6, 6'), 139.9, 139.5, 139.3 (Galloyl C-1, 1', 1''), 115.9, 115.6 (HHDP C-1, 1'), 110.1, 110.0, 109.2 (Galloyl C-2, 2', 2'', 6, 6', 6''), 107.9, 107.7 (HHDP C-3, 3'), 93.5 (glucose C-1), 73.2 (glucose C-3), 72.8 (glucose C-5), 71.7 (glucose C-2), 70.6 (glucose C-4), 63.0 (glucose C-6).

Cornusiin A (**21**): An off-white amorphous powder. HR-ESI-MS *m/z*: 1569.1617 ([M-H]$^-$, Calcd. for $C_{68}H_{50}O_{44}$-H: 1569.1602). ^{1}H-NMR (500 MHz, acetone-d_6 + D_2O) δ: 7.08, 7.07, 7.02, 7.012, 7.007, 6.99, 6.98, 6.95 (each s, 5H in total, Val Hc, 2 × Galloyl-H), 6.948, 6.88, 6.86, 6.84 (each s, 2H in total, Galloyl-H), 6.66, 6.644, 6.641, 6.63, 6.613, 6.611, 6.60, 6.59 (each s, 2H in total, HHDP-H, Val H_A), 6.50, 6.49, 6.48, 6.47 (each s, 1H in total, HHDP-H), 6.22, 6.181, 6.176, 6.171 (each s, 1H in total,Val H_B), 5.79 (t, *J* = 9.9 Hz, glucose H-3_L of α_L-β_R form), 5.785 (t, *J* = 9.9 Hz, glucose H-3_L of α_L-α_R form), 5.67 (t, *J* = 9.9 Hz, glucose H-3_R of β_L-β_R form), 5.66 (t, *J* = 9.9 Hz, glucose H-3_R of α_L-α_R form), 5.50 (d, *J* = 3.5 Hz, glucose H-1_R of β_L-α_R form), 5.48 (d, *J* = 2.5 Hz, glucose H-1_R of α_L-α_R form), 5.45-4.96 (complicated peaks), 4.77-4.74, 4.60-4.57, 4.40-4.36, 4.19-4.14 (m, glucose H-5_L and H-5_R of the four forms), 4.48 (1H, d, *J* = 8.0 Hz, glucose H-1_L of α_L-β_R form), 4.45 (1H, d, *J* = 8.0 Hz, glucose H-1_L of β_L-β_R form), 3.93-3.74 (m, glucose H-6_L and H-6_R of the four forms).

Rugosin D (**22**): A light-brown amorphous powder. HR-ESI-MS *m/z*: 1897.1763 ([M+Na]$^+$, Calcd. for $C_{82}H_{58}O_{52}$+Na: 1897.1787). ^{1}H-NMR (500 MHz, acetone-d_6 + D_2O) δ: 7.09 (1H, s, Val-H_C), 7.07, 6.984, 6.979, 6.97, 6.93 (each 2H, s, Galloyl-H), 6.61 (1H, s, HHDP-H_B), 6.47 (1H, s, Val H_A), 6.46 (1H, s, HHDP-H_A), 6.15 (1H, s, Val H_B), 6.12 (1H, d, *J* = 8.5 Hz, glucose I H-1), 6.02 (1H, d, *J* = 8.5 Hz, glucose II H-1), 5.78 (1H, t, *J* = 9.5 Hz, glucose I H-3), 5.74 (1H, t, *J* = 9.5 Hz, glucose II H-3), 5.57 (1H, dd, *J* = 8.5, 9.5 Hz, glucose I H-2), 5.52 (1H, dd, *J* = 8.5, 9.5 Hz, glucose II H-2), 5.27-5.20 (2H, m, glucose I H-6, glucose II H-6), 5.14 (1H, t, *J* = 9.5 Hz, glucose I H-4), 5.12 (1H, t, *J* = 9.5 Hz, glucose II H-4), 4.47 (1H, m, glucose I H-5), 4.40 (1H, m, glucose II H-5), 3.78 (overlapped, glucose I H-6, glucose II H-6). ^{13}C-NMR (126 MHz, acetone-d_6 + D_2O) δ: 168.3 (HHDP C-7'), 168.0 (Val C-7'), 167.9 (Val C-7), 167.8 (HHDP C-7), 166.69, 166.66, 166.2, 166.1, 165.2 (Galloyl C-7, 7', 7'', 7''', 7''''), 162.3 (Val C-7''), 146.6 (Val C-4'), 146.0, 145.88, 145.87, 145.74 145.7 (Galloyl C-3, 3', 3'', 3''', 3'''', 5, 5', 5'', 5''', 5''''), 145.2, 145.12, 145.11 (HHDP C-4, 4', Val C-4), 145.0, 144.6 (Val C-6, 6'), 144.3 (2C, HHDP C-6, 6'), 143.2 (Val C-5''), 141.4 (Val C-4''), 140.3 (Val C-3''), 139.9, 139.6, 139.5, 139.29, 139.27 (Galloyl C-4, 4', 4'', 4''', 4''''), 137.8 (Val C-2''), 137.0 (Val C-5'), 136.58, 136.55 (HHDP C-5, Val C-5), 136.2 (HHDP C-5'), 126.1, 125.6, 125.4, 125.1 (HHDP C-2, 2', Val C-2, 2'), 119.92, 119.0, 119.7, 119.6, 119.2 (Galloyl C-1, 1', 1'', 1''', 1''''), 117.6 (Val C-1'), 115.9, 115.6 (HHDP C-1, Val C-1), 115.5 (HHDP C-1'), 112.3 (Val C-1''), 110.1, 110.07, 110.05, 110.01, 109.9 (Galloyl C-2, 2', 2'', 2''', 2'''', 6, 6', 6'', 6''', 6''''), 109.7 (Val C-6''), 108.0 (HHDP C-3'), 107.7, 107.6 (HHDP C-3, Val C-3), 104.6 (Val C-3'), 93.5 (glucose I C-1), 93.0 (glucose II C-1), 73.3 (2C, glucose I C-3, II C-3), 72.8, 72.7 (glucose I C-5, II C-5), 71.6 (2C, glucose I C-2, II C-2), 70.6, 70.5 (glucose I C-4, II C-4), 63.0, 63.9 (glucose I C-6, II C-6).

(7′*S*,8′*R*)-Dihydrodehydrodiconiferyl alcohol-9′-*O*-β-D-glucose (**23**): A pale brown amorphous powder. HR-ESI-MS *m/z*: 545.1974 ([M+Na]$^+$, Calcd. for $C_{26}H_{34}O_{11}$+Na: 545.1993). CD (MeOH) [*α*] (nm) -1.1 × 10^3 (220), +1.0 × 10^4 (241), +3.5 × 10^3 (292), ^{1}H-NMR (500 MHz, MeOH-d_4) δ: 6.98 (1H, d, *J* = 2.0 Hz, H-2'), 6.85 (1H, dd, *J* = 2.0, 8.0 Hz, H-6'), 6.79 (1H, br s, H-6), 6.75 (1H, d, *J* = 8.0 Hz, H-5'), 6.71 (1H, br d, *J* = 1.5 Hz, H-2), 5.57 (1H, d, *J* = 6.5 Hz, H-7'), 4.34 (1H, d, *J* = 8.0 Hz, glucose H-1), 4.10 (1H, dd, *J* = 8.0, 10.0 Hz, H-9'), 3.88-3.83 (2H, m, H-9', glucose H-6), 3.84 (3H, s, 3-OCH_3), 3.82 (3H, s, 3'-OCH_3) 3.70-3.62 (2H, m, H-8', glucose H-6), 3.55 (2H, t, *J* = 6.5 Hz, H-9), 3.36 (1H, t, *J* = 8.5 Hz, glucose H-3), 3.31-3.26 (2H, m, glucose H-4, 5), 3.22 (1H, dd, *J* = 8.0, 8.5 Hz, glucose H-2), 2.62 (2H, t, *J* = 6.5 Hz, H-7), 1.82 (2H, m, H-8). ^{13}C-NMR (126 MHz, MeOH-d_4) δ: 149.0 (C-3''), 147.5 (C-4), 147.4 (C-4'), 145.2 (C-3), 137.0 (C-1), 134.7 (C-1'), 129.7 (C-5), 119.8 (C-6'), 118.2 (C-6), 116.1 (C-5'), 114.2 (C-2), 110.8 (C-2'), 104.3 (glucose C-1), 89.2 (C-7'), 78.2 (glucose C-3), 78.1 (glucose C-5), 75.2 (glucose C-2), 72.3 (C-9'), 71.7 (glucose C-4), 62.8 (glucose C-6), 62.2 (C-9), 56.8 (3-OCH_3), 56.5 (3'-OCH_3), 52.9 (C-8'), 35.8 (C-8), 32.9 (C-7).
